# An open‐label trial of cryopreserved human umbilical cord in the treatment of complex diabetic foot ulcers complicated by osteomyelitis

**DOI:** 10.1111/wrr.12754

**Published:** 2019-08-19

**Authors:** William A. Marston, John C. Lantis, Stephanie C. Wu, Aksone Nouvong, Tommy D. Lee, Nicholas D. McCoy, Herbert B. Slade, Scheffer C. Tseng

**Affiliations:** ^1^ Division of Vascular Surgery University of North Carolina School of Medicine Chapel Hill North Carolina; ^2^ Department of Surgery Mt Sinai West and St Luke's Hospitals New York New York; ^3^ Department of Podiatric Surgery & Applied Biomechanics Rosalind Franklin University North Chicago Illinois; ^4^ Department of Surgery UCLA Los Angeles California; ^5^ Research & Development TissueTech, Inc. Miami Florida; ^6^ Department of Pediatrics University of North Texas Health Sciences Center Fort Worth Texas

## Abstract

Clinical trials of potential new therapies for diabetic foot ulcers rarely enroll patients whose wounds extend to muscle, fascia, or bone with clinical and radiographic evidence of underlying osteomyelitis. An open‐label, multicenter trial of cryopreserved human umbilical cord (TTAX01) was undertaken in 32 subjects presenting with such complex wounds with a mean duration of 6.1 ± 9.0 (range: 0.2–47.1) months and wound area at screening of 3.8 ± 2.9 (range: 1.0–9.6) cm^2^. Aggressive surgical debridement at baseline resulted in 17 minor amputations and an increase in mean wound area to 7.4 ± 5.8 (range: 1.1–28.6) cm^2^. All subjects were placed on systemic antibiotics for at least 6 weeks in conjunction with baseline application of TTAX01. Repeat applications were made at no less than 4‐week intervals over the 16‐week trial. Initial closure occurred in 18 of 32 (56%) wounds, with 16 (50%) of these having confirmed closure in 16 weeks with a median of one‐product application. Cases with biopsy confirmed osteomyelitis (*n* = 20) showed initial closure in 12 (60%) wounds and confirmed closure in 10 (50%) wounds. Four of the five ulcers presenting as recurrences experienced confirmed closure. Mean overall time to healing was 12.8 ± 4.3 weeks. Mean wound area reduction from baseline was 91% for all wounds. Of the 16 wounds without confirmed closure during the 16‐week treatment period, five (31.3%) achieved 99–100% wound area reduction by their final visit. The product was well tolerated. Two minor amputations occurred during the study period due to recurrent or persistent osteomyelitis; however, there were no major amputations.

## INTRODUCTION

Diabetes is a significant public health challenge affecting more than 30 million people in the United States alone, a figure that has increased sixfold since 1980.^1^ If this trend continues, as many as one in three US adults could have diabetes by 2050. Patients with diabetes face a number of health concerns including peripheral sensory neuropathy and development of diabetic foot ulcers (DFU). It is estimated that 25% of people with diabetes will develop a foot ulcer in their lifetime,^2^ and delayed wound healing of these ulcers is the single most common cause of lower extremity amputation among this population.^1^, ^3^ Complex ulcers exhibiting exposed bone, tendon, muscle, and/or joint capsule are at particularly high risk for infection‐related ulcer complications such as osteomyelitis,^2^, ^4^ which has been shown to increase amputation risk fourfold when compared to soft tissue infection alone.^5^, ^6^ This is alarming given the 5‐year survival rate after one major lower extremity amputation is estimated to be as low as 50%.^7^, ^8^

The current standard of care (SOC) regimen for complex DFU involves surgical debridement of necrotic tissue and bone, prevention or control of infection with antibiotics, and offloading. However, the rate of wound healing with the current SOC is poor, with only 30% of DFUs healing within 12 weeks and 45% healing regardless of the time period.^9^ This healing rate is even lower for complex wounds, with 33% achieving complete wound closure at 20 weeks.^10^ The nonhealing nature of these chronic wounds has led to the development of advanced cellular and tissue‐based products and therapeutic biologics to move these wounds from a chronic, stalled condition to a trajectory of healing. However, none of these treatment options are specifically intended to promote healing of wounds with exposed bone, tendon, muscle, and/or joint capsule complicated with osteomyelitis. These conditions are typically excluded in clinical trials, such as with Apligraf®, Dermagraft®, and Oasis® wound matrix.^11^, ^12^, ^13^, ^14^, ^15^ In addition, only a limited number of studies support the use of hyperbaric oxygen therapy for ulcers complicated by ischemia but not for well‐perfused deep ulcers complicated by osteomyelitis.^16^, ^17^, ^18^ Negative pressure wound therapy (NPWT) is another option for these cases, especially when combined with wide debridement or partial amputation.^19^, ^20^, ^21^ Therapy may continue to complete closure or may be stopped after complete granulation to allow surgical closure or healing by secondary intent. Thus, there is a dire clinical need for a novel biologic treatment to improve and accelerate healing of complex, nonhealing DFUs complicated by osteomyelitis in combination with surgical resection and systemic antibiotic therapy.

Amniotic membrane (AM) and umbilical cord (UC) tissue have long been recognized to exert unique anti‐inflammatory, anti‐scarring, and pro‐regenerative properties in various indications including dermal wounds.^22^, ^23^, ^24^ A protein matrix component found in these tissues, heavy chain‐hyaluronan/pentraxin 3 (HC‐HA/PTX3), is believed to promote resolution of inflammatory processes, support stem cells, and inhibit myofibroblasts such that scarring is reduced or absent in healed tissues.^25^, ^26^, ^27^, ^28^, ^29^ As such, these tissues can promote wound healing at the cellular level and are suitable for use as a biological membrane without engraftment.^25^ Although there are several processing methods available for AM and UC tissues, cryopreservation using a patented process (CryoTek®, TissueTech, Inc., Miami, FL) has been shown to preserve the active HC‐HA/PTX3 matrix component significantly better than dehydration.^30^ Moreover, when compared to AM, UC contains significantly higher amounts of high‐molecular‐weight hyaluronic acid (HA) and HA‐containing active matrix component.^30^ The regenerative therapeutic effect of human cryopreserved UC (cUC) in the repair of skin defects associated with bone exposure is reported in several animal models of spina bifida.^31^, ^32^ In these models, cUC results in regeneration of epidermal, dermal, and subdermal components with minimal inflammatory cell migration.^31^ In comparison with acellular dermal matrix where there was an equal amount of cellular ingrowth, there was decreased acute inflammation and more organized cellular ingrowth of epidermal and meningeal cells in the human cUC.^32^ Preliminary studies using the commercially available UC product NEOX™ CORD 1K (Amniox Medical, Inc., Miami, FL) further suggest cUC is safe and useful in promoting healing of complex foot ulcers with osteomyelitis, with healing rates reported from 79 to 100% when combined with negative pressure therapy.^33^, ^34^ TTAX01, an investigation biologic form of the cUC made under Good Manufacturing Practices with additional validations and potency measurements, also contains these potentially beneficial properties. This open‐label pilot study was designed to estimate the efficacy of TTAX01 plus standard care (SC) in achieving complete wound closure of complex nonhealing DFU. A secondary purpose was to examine the operational aspects of the protocol in advance of a phase 3 pivotal trial using TTAX01, including ease of compliance with various decision points.

## METHODS AND PATIENTS

### Trial design and participants

**Figure 1:**
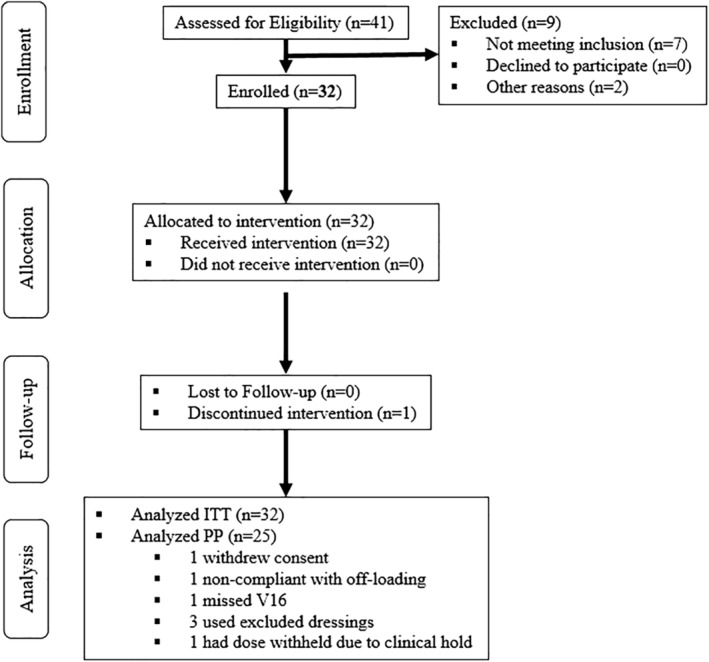
CONSORT flow diagram.

This was a multicenter, open‐label trial conducted at healthcare facilities in the United States (11 centers), with each center enrolling at least one patient over a 10‐month period beginning October 2017 (NCT03230175). Initial debridement procedures took place at a mix of inpatient and outpatient settings, with all follow‐up visits conducted in outpatient settings. The protocol received Institutional Review Board approval for each participating center. One minor protocol amendment was made before enrolling the third patient, providing clarifications and refinements including allowance of sutures or staples, and fenestration after fixation of the tissue, as well as increasing the number of potential sites to 12. The goal for enrollment was 30–36 to obtain a good estimate of the mean response rate, based on the central limit theorem. The main study inclusion criteria were as follows: ≥18 years of age; confirmed diagnosis of type I or II diabetes mellitus; index ulcer area ≥ 1.0 cm^2^ and ≤10.0 cm^2^; exposure of tendon, muscle, joint capsule or bone; positive probe to bone test; radiographic evidence of osteomyelitis; adequate perfusion as showed by dorsum transcutaneous oxygen test (TcPO_2_) ≥ 40 mmHg, ankle‐brachial index (ABI) ≥ 0.7, or great toe pressure ≥ 50 mmHg. Wound duration was not limited. Subjects were excluded if they had a HbA1c > 12%, serum albumin ≤ 2.0 g/dL, were currently taking medications that could impair healing, or had a malignancy other than non‐melanoma skin cancer within 5 years. Wounds on the dorsum of the foot, and women who were pregnant or lactating were also excluded. Each of the 11 sites enrolled one to four subjects. A total of 41 patients were screened, with 32 enrolled and included in the intention to treat (ITT) population. Of these 32 subjects, one withdrew consent, one was non‐compliant, one missed visit 16, one had dose withheld due to clinical hold, and three used excluded dressings. Thus, the per protocol (PP) population comprised 25 participants (Figure 1). The trial ran to completion as planned.

### TTAX01

TTAX01 is a cryopreserved human UC product derived from donated human placental tissue following healthy, live, caesarian section, full‐term births after determination of donor eligibility, and placenta suitability. TTAX01 is manufactured by TissueTech Inc. utilizing the proprietary CRYOTEK® process, which devitalizes the living cells but retains the natural structural and biological characteristics relevant to this tissue. TTAX01 is aseptically processed in compliance with both current Good Tissue Practices and Good Manufacturing Practices.

### Interventions

Aggressive surgical debridement, including minor amputations, was carried out in an operating room as the initial procedure to remove all infected tissue including infected bone, during which time tissue and bone biopsies were obtained to further confirm the presence of osteomyelitis. Once debridement was completed, TTAX01 was applied to the open wound surface and fixed with sutures or staples, then fenestrated in place and covered using the investigator's choice of secondary dressing. Subjects were started or continued on empirically chosen systemic antibiotics for a total of 6 weeks, which could be adjusted based on culture and sensitivity results from the biopsy specimens. Sutures/staples were removed when no longer needed. TTAX01 could be applied at ≥4 weeks following any previous application, only if the wound was not showing evidence of healing. Appropriate sharp debridement was performed weekly. Off‐loading consisted of a device appropriate to the location of the wound, preferably using a full length boot or total contact cast unless not appropriate, in which case a substitute off‐loading device could be used per the physician's suggestion with sponsor approval. Subjects attended weekly evaluation visits over 16 weeks or until the wound was 100% re‐epithelialized without drainage. Complete wound closure was confirmed at two additional visits each 2 weeks apart. The wound had to remain closed for this 4 week period to qualify as a confirmed closure. Evaluation of wounds included image capture and electronic measurement via automatic tracing of area (cm^2^), depth (cm), and volume (cm^3^) using the eKare inSight™ measuring device (eKare Inc., Fairfax, VA). When a wound was judged to be closed, four additional images were captured at 90, 180, 270, and 360° to avoid ambient light reflection causing difficulty for the blinded reviewer. Sites were extensively trained on the proper use of the imaging device.

### Endpoints

The primary efficacy endpoint was the proportion of subjects with confirmed wound closure during the 16‐week treatment period, determined through direct examination by the investigator and blinded review of photographs by a third party dermatologist with expertise in advanced wound care. Secondary efficacy endpoints included time to closure, percentage change in wound area at each visit from baseline, and proportion of wounds with confirmed osteomyelitis achieving complete closure. Safety endpoints included proportion with ulcer‐related complications (infection, recurrent osteomyelitis, and gangrene) and proportion undergoing amputations. Spontaneous and elicited adverse events were evaluated for trends and possible association with treatment.

### Statistical analysis

All continuous data were expressed as mean ± standard deviation (range), whereas categorical variables were expressed as frequency and percentages. The primary efficacy analysis was performed for the ITT population, which included all enrolled subjects. The PP dataset removed subjects with major protocol violations and those for whom the primary endpoint could not be adequately evaluated (Figure 1). The non‐parametric Cochran–Mantel–Haenszel test with adjustment for site was used to examine the proportion of subjects with complete wound closure over the 16‐week treatment period between two post‐debridement ulcer size groups (those ≤ mean wound area and those > mean wound area at baseline). Cox regression, ANCOVA, and Cochran–Mantel–Haenszel tests were used in the analysis of secondary endpoints. A *p* value less than 0.05 was considered statistically significant.

## RESULTS

Demographics and baseline characteristics for all 32 subjects are summarized in Table 1. The average wound duration prior to TTAX01 application was 6.1 ± 9.0 months. Osteomyelitis was diagnosed at baseline according to positive probe to bone and positive radiographic evidence in all wounds and was confirmed by bone biopsy in the majority of subjects (63%). A small percentage (6%) had only a positive microbial/fungal culture at baseline without biopsy confirmation of osteomyelitis. In the remaining cases, neither infection nor osteomyelitis was confirmed via biopsy, although it is noted that 18 of the 32 subjects were receiving systemic antibiotics before the collection of material for microbiologic testing. The initial procedure debridement to remove infected and necrotic bone resulted in 17 minor amputations, and an average wound area increase of 200%. One subject withdrew consent and left the study after 2 weeks. Otherwise, all subjects continued in the study until their wound was closed, or they had reached the end of the 16‐week treatment period.

**Table 1:** Demographics and baseline characteristics

	TTAX01 treatment (*N* = 32)
Age (years)	57.7 ± 10.2 (41.0–73.0)^a^
Gender	
Female	1 (3%)
Male	31 (97%)
Race	
Alaskan Native/American Indian	1 (3%)
Black/African American	8 (25%)
White/Caucasian	22 (69%)
Other	1 (3%)
Ethnicity	
Hispanic/Latino	8 (25%)
Not Hispanic/Latino	24 (75%)
Occurrence	
First occurrence	27 (84%)
Reoccurrence	5 (16%)
Osteomyelitis	
Positive tissue culture	22 (689%)
Confirmed bone infection	20 (63%)
Wound area (cm^2^)	7.4 ± 5.8 (1.1–28.6)
Wound duration (months)	6.1 ± 9.0 (0.2–47.1)
Wound location	
Heel	5 (16%)
Lateral surface	3 (9%)
Medial surface	2 (6%)
Plantar surface	18 (56%)
Toes (interdigital)	4 (13%)

a Values are reported as mean ± SD (min–max) or number (%).

The product was safe and well tolerated as used in this study. There were no local or systemic adverse events attributable to the product. One small laceration occurred as a result of a suture pulling through skin, and one initial application became dislodged, requiring re‐application at the subsequent visit. There were two minor amputations during the study because of persistent or recurrent osteomyelitis. There were no major amputations.

**Figure 2:**
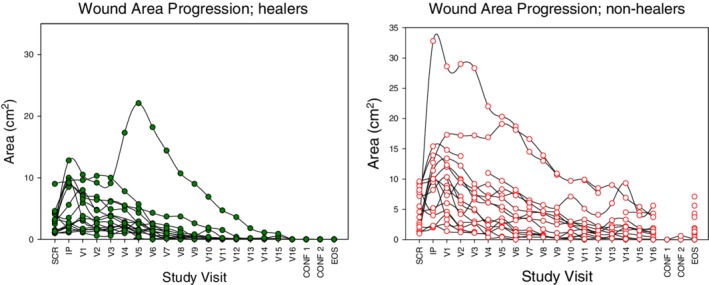
Individual subjects wound area by visit week. CONF1 and CONF2 are confirmation of healing visits. EOS is the end of study visit.

In the ITT analysis, 16 of 32 (50%) subjects achieved confirmed wound healing within 16 weeks (Figure 2) with an average of 1.5 ± 0.8 applications (median of 1, range 1–3). of TTAX01 being sufficient to achieve healing. Those which did not heal received an average of 2.3 ± 1.1 (median of 2, range 1–4) applications. Removing two sites which had only a single subject, the median number of applications across the remaining nine sites was normally distributed (Shapiro–Wilk W‐Statistic = 0.899, *p* = 0.195).

Among the five subjects presenting with recurrent wounds, four (80%) achieved confirmed healing. Ten of 20 (50%) subjects with biopsy confirmed osteomyelitis healed within 16 weeks. The mean time to achieve closure was 12.8 ± 4.3 weeks. Cox regression analysis found that ulcer duration significantly affected the time needed to heal (*p* < 0.02). Logistic regression similarly revealed a significant relationship (*p* < 0.05) between log‐transformed wound duration and healing, with older wounds being more likely to heal. Interpretation is difficult, given that wound duration and duration of osteomyelitis were not necessarily identical, wounds were not required to be chronic, and aggressive baseline debridement removed any chronic wound tissue. When controlling for ulcer duration, baseline wound area was not a statistically significant covariate for healing by week 16. For weeks 8 through 16, smaller than average wounds achieved significantly higher proportions of wound closure when compared to larger than average wounds (*p* < 0.05).

**Figure 3:**
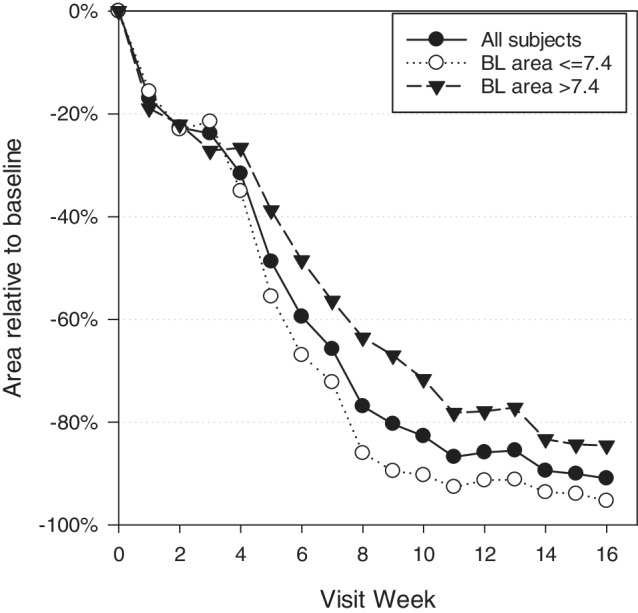
Percentage change in wound area from baseline. Plots of the average change all subjects, and for those whose wounds were above or below the average baseline wound area of 7.4 cm^2^.

**Figure 4:**
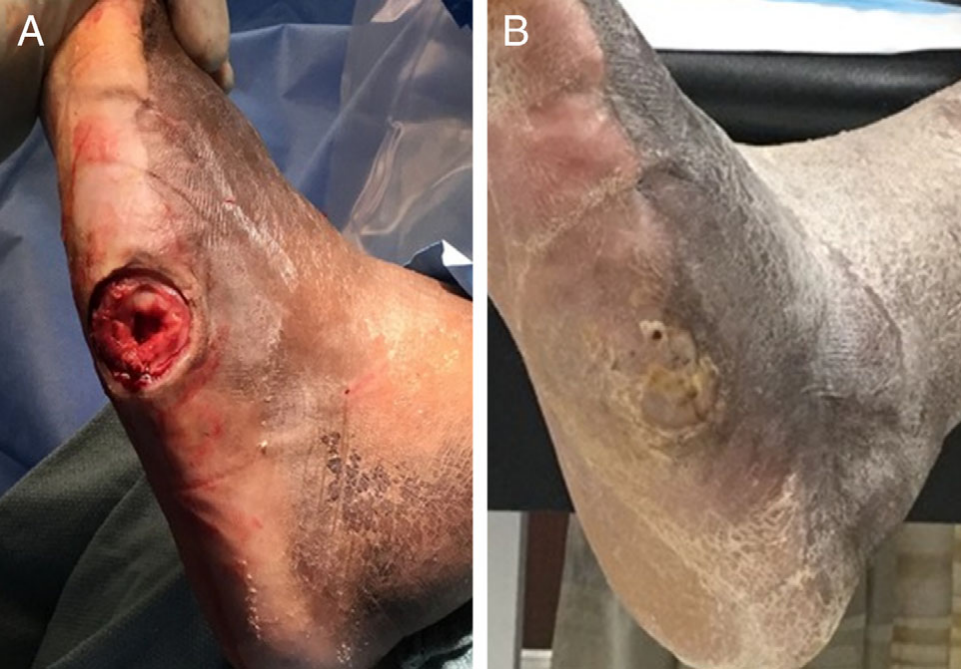
Representative case of successful wound closure with one application of TTAX01. Subject presented with a 9.0 cm^2^ wound at screening, which slightly increased to 9.1 cm^2^ following initial debridement (A). The wound achieved complete closure by visit 12 using one application of TTAX01 (B).

**Figure 5:**
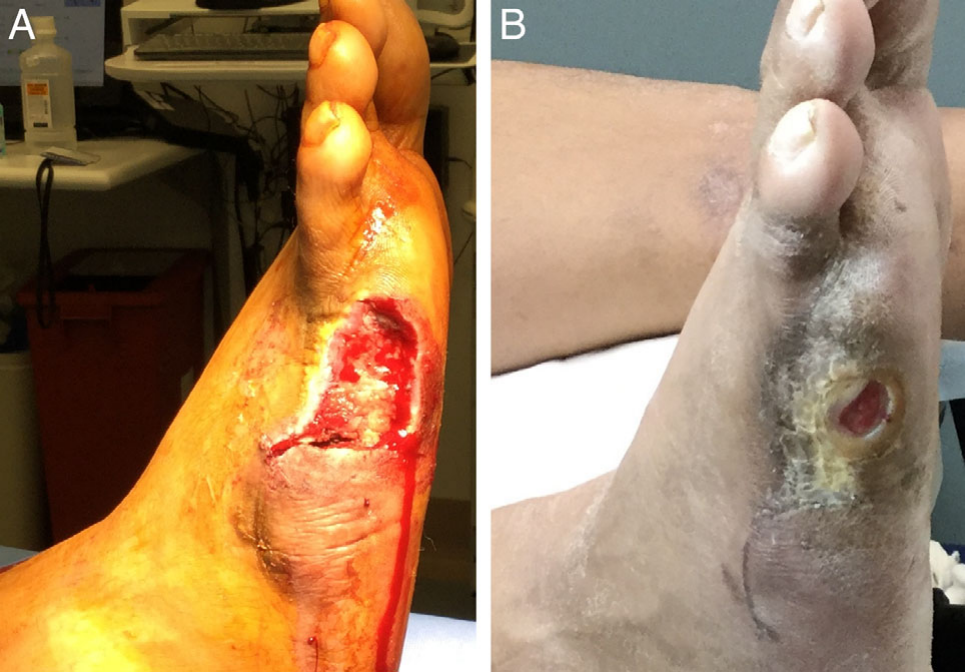
Representative case of failed wound closure by visit 16. Subject presented with a 7.2 cm^2^ wound at screening. Following initial debridement (A), wound measured 8.2 cm^2^. The subject received a second application of TTAX01 at study visit 11 due to stalled healing. By final visit 16 (B), wound showed an 89% reduction from baseline area and measured 0.9 cm^2^.

Over the treatment period, the mean reduction from baseline in wound surface area was 91% for all subjects, with smaller than average wounds closing faster than those larger than the mean (Figure 3). Initial but unconfirmed closure was seen in two additional subjects, whereas one other closed at the exit visit and two more showed wound area reduction of ≥99% at the exit visit. In the per protocol analysis, 15 of 25 (60%) wounds achieved confirmed healing. One representative case of wound each that did and did not achieve wound closure during the 16‐week study period is presented in Figures 4 and 5, respectively.

## DISCUSSION

The results of this multicenter, open‐label, pilot study suggest that TTAX01 is a promising therapy for the management of complex, nonhealing DFUs complicated by osteomyelitis. Following TTAX01 treatment, the overall healing rate was favorable compared to the US Wound Registry standard of care success rates, which reported 31% healing of all DFUs at 12 weeks and 45% healing regardless of time period.^9^ SOC treatment success is reported to be even lower for complex wounds, with only 33% achieving complete wound closure at 20 weeks.^10^ As a further point of reference, the chronic wounds database from Healogics Inc. was accessed to examine healing rates in patients matched against the inclusion criteria for this study. Among 6,498 matching records in patients with diabetic Wagner 3 or 4 ulcers, the healing rate at 16 weeks was 34.7% using all available therapies. Thus, the results of the present study are encouraging given the unmet medical need of a treatment for complex wounds complicated with osteomyelitis, a population exhibiting high morbidity and mortality.

Although a number of advanced therapies have been developed to improve wound healing of DFUs, none of these products are indicated to treat complex wounds or wounds with osteomyelitis.^11^, ^12^, ^13^, ^14^, ^15^ Only Theraskin™ carries an indication for use in exposed tendon, joint, and/or bone in a reported clinical case study^35^ but not a prospective, randomized, controlled clinical trial. In one study of 188 patients, only 2% of ulcers extended to the tendon, and less than 1% of ulcers had exposed bone.^35^ In addition, Theraskin is not indicated for ulcers with severe infection including osteomyelitis. Aside from the present study, successful use of cUC in treating complex DFUs with osteomyelitis has been reported in two retrospective studies.^33^, ^34^ In the first study,^33^ 26 of 33 (79%) wounds achieved complete wound closure in 16 ± 9.3 (range: 4–44) weeks with 1.2 ± 0.4 cUC applications. The average wound size was 16 ± 18 cm^2^. In the second study,^34^ all 14 wounds (33 ± 22 cm^2^) achieved complete re‐epithelialization. In the present study, 60% of wounds achieved complete wound closure in 12.8 ± 4.3 weeks with TTAX01, a result that is compatible with the healing rates observed in the two aforementioned retrospective studies using a commercially available form of cUC (NEOX CORD 1K).

HC‐HA/PTX3 is an active matrix component that may contribute to TTAX01's clinical efficacy through its known anti‐inflammatory, anti‐scarring, and pro‐regenerative effects.^25^, ^26^, ^27^, ^28^, ^29^ HC‐HA/PTX3 promotes apoptosis and phagocytosis of activated neutrophils, polarizes macrophages toward the M2 phenotype, and increases anti‐inflammatory cytokine expression.^26^, ^28^, ^36^ Polarization of macrophages is especially important as the lack of transition from M1 to M2 macrophages is a hallmark of nonhealing cutaneous wounds.^37^, ^38^ In addition, AM and UC tissues exert a direct anti‐scarring effect by suppressing TGF‐β signaling at the transcriptional level.^22^, ^25^ Additional studies suggest that this modulation of TGF‐β signaling favors keratinocyte proliferation and migration that can allow chronic wounds to progress from their non‐healing state into re‐epithelialization.^39^ In addition to the observed anti‐inflammatory and anti‐scarring effects, HC‐HA/PTX3 has also been shown to maintain quiescence of stem cells in the corneal limbal niche,^29^, ^40^ suggesting its clinical usefulness in expanding the stem cell pool to promote regenerative healing in wounds that are under the threat of non‐resolving inflammation. Collectively, these actions may provide an optimal healing environment to promote re‐epithelialization in complex DFUs.

The applicability of the results is limited to the estimation of response rate in subsequent trials of similar design. The reported study is one of several to be conducted in the clinical development of this investigational new biologic. The encouraging findings in this study require confirmation in larger studies involving randomized comparison to other treatment strategies for patients with complex nonhealing DFUs.
